# Origin and Function of Structural Diversity in the Plant Specialized Metabolome

**DOI:** 10.3390/plants10112393

**Published:** 2021-11-06

**Authors:** Sandrien Desmet, Kris Morreel, Rebecca Dauwe

**Affiliations:** 1 Department of Plant Biotechnology and Bioinformatics, Ghent University, 9052 Gent, Belgium; sandrien.desmet@psb.vib-ugent.be (S.D.); kris.morreel@ugent.be (K.M.); 2 Center for Plant Systems Biology, VIB, 9052 Gent, Belgium; 3 Unité de Recherche Biologie des Plantes et Innovation (BIOPI), UMR Transfrontalière BioEcoAgro, Université de Picardie Jules Verne, 80000 Amiens, France

**Keywords:** plant specialized metabolome, biosynthesis pathways, metabolite modification, structural diversity, structure-function relationship

## Abstract

The plant specialized metabolome consists of a multitude of structurally and functionally diverse metabolites, variable from species to species. The specialized metabolites play roles in the response to environmental changes and abiotic or biotic stresses, as well as in plant growth and development. At its basis, the specialized metabolism is built of four major pathways, each starting from a few distinct primary metabolism precursors, and leading to distinct basic carbon skeleton core structures: polyketides and fatty acid derivatives, terpenoids, alkaloids, and phenolics. Structural diversity in specialized metabolism, however, expands exponentially with each subsequent modification. We review here the major sources of structural variety and question if a specific role can be attributed to each distinct structure. We focus on the influences that various core structures and modifications have on flavonoid antioxidant activity and on the diversity generated by oxidative coupling reactions. We suggest that many oxidative coupling products, triggered by initial radical scavenging, may not have a function *in se*, but could potentially be enzymatically recycled to effective antioxidants. We further discuss the wide structural variety created by multiple decorations (glycosylations, acylations, prenylations), the formation of high-molecular weight conjugates and polyesters, and the plasticity of the specialized metabolism. We draw attention to the need for untargeted methods to identify the complex, multiply decorated and conjugated compounds, in order to study the functioning of the plant specialized metabolome.

## 1. Introduction

With the development of new metabolomics techniques, the improvement of detection methods and, in parallel, the ever-expanding number of metabolomics profiling studies performed on different plant species or plants subjected to different treatments or mutations, the number of known specialized plant metabolites is continuously increasing, hence leading to the impression that the number of different structures is infinite [1,2,3,4,5,6,7,8]. An aspect of plant biology that is not understood yet is the benefit of this huge structural diversity for plants. It is widely accepted today that specialized metabolites contribute to the chemical defense of plants, as sessile organisms without an adaptive immune system, against different forms of biotic and abiotic stress [9,10]. In that context, it could be tempting to pursue a specific biological function for each distinct metabolite. Certain metabolites seem, indeed, to be the result of a co-evolution with an acceptor, and others play an obvious role as UV-screen or antioxidant [11,12,13,14]. The principles of evolution tell us that wasteful biosynthetic pathways will be selected against and are prone to disappear, whereas each addition to a biosynthetic pathway can only be maintained by selection if it enhances the fitness of the plant [15]. Logically, such constraints should lead to a rather limited number of optimized defense compounds. The plethora of structurally similar yet distinct compounds found in each plant species’ specialized metabolome seems, in that context, to form an evolutionary inconsistency. The “Screening Hypothesis”, which was first described in 1991, assumes that most specialized metabolites produced by a plant do not possess biological activity [16]. According to this theory, natural selection favored plants that produce the largest possible chemical diversity, at the lowest possible cost. The greater the chemical diversity of the specialized metabolite pool, the greater the chance that it contains a useful, biologically active compound that can protect the plant. The main tool to obtain maximal structural diversity at minimal cost consists of matrix grid biosynthetic pathways in which a limited number of enzymes with a broad substrate tolerance participate in the production of a high diversity of compounds [17]. Well-known examples of such grid biosynthetic pathways are those leading to the production of carotenoids, anthocyanins, monolignols and glucosinolates. The “Screening Hypothesis” sees the specialized metabolome mainly as a plant defense system against different types of stress [18]. In contrast, another hypothesis, which was formulated twenty years later, states that the first and major role of the specialized metabolome lies in its participation in the redox chemistry, particularly the reduction in reactive oxygen species (ROS) and, in the absence of ROS, the reduction in molecular oxygen to H_2_O_2_ by the exact same compounds [18]. This hormetic effect (antagonistic effects of low and high doses) in the redox chemistry is seen as an essential characteristic of the specialized metabolome, making it involved in both regulatory and destructive processes: low levels of ROS stimulate growth, cell differentiation and stress resistance, whereas high levels induce programmed cell death [18]. Mainly phenolics, but also terpenoids and alkaloids, function as electron donors or acceptors in electron transfer reactions, and often possess both pro- and antioxidant activity, depending on their concentration and the redox environment. The high structural diversity among metabolites undergoing similar redox reactions suggests that the three-dimensional structure of the carbon skeleton does not determine the redox activity, but that these reactions are enabled by the presence of specific functional groups. It has therefore been proposed that a primary function in the redox chemistry of specialized metabolites is in accordance with weak selective constraints on the carbon structure, and high structural diversity as a result [18].

To help readers form an opinion on the above, seemingly contradicting theories, we review here three main sources of structural variation in the plant specialized metabolome. First, we describe the major pathways, each starting from a few distinct primary metabolism precursors and leading to four distinct basic carbon skeleton core structures: polyketides and fatty acid derivatives, terpenoids, alkaloids and phenolics. Second, we address the structural diversity due to modifications to the core skeleton, such as changes in hydrogenation level, hydroxylations and methoxylations, specifically for flavonoids, and we discuss the impact of these modifications on flavonoid antioxidant activity. It should be noted here that, in other classes of specialized metabolites, other modifications of the core skeleton, such as the cyclization or ring cleavage, the addition or removal of skeletal atoms or bond migration, are observed but will not be addressed here. Third, we address the role of so-called “decorative” reactions, conjugations and polymerizations in the plant specialized metabolome. Interpreting the results of recent metabolite profiling studies in wild type, mutant and transgenic plants, we show how the combination of substrate promiscuity of enzymes, multiple decorations and oxidative coupling can lead to a seemingly endless variation in structures in the plant specialized metabolome.

## 2. The Four Major Specialized Metabolome Pathways

### 2.1. Polyketides and Fatty Acid Derivatives

**Figure 1:**
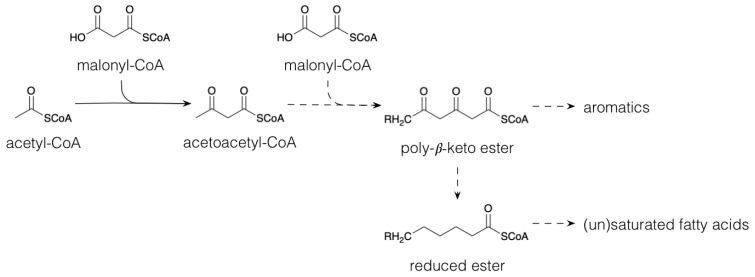
Biosynthesis of polyketides and (un)saturated fatty acids. Dashed arrows indicate multiple conversions.

Polyketides are a large group of secondary metabolites that result from the condensation of acetyl-CoA (C_2_) with one or more Coenzyme A (CoA) esters, such as malonyl-CoA (C_3_) via chain extension [19] (Figure 1); a process that is catalyzed by polyketide synthases [20]. By losing its carboxyl group, malonyl-CoA condensation leads to a C_2_-unit extension. Different reduction steps generate the myriad of saturated and unsaturated fatty acids in plants. In higher plants, C18 polyunsaturated fatty acids (PUFAs), mainly linoleic acid and α-linolenic acid, are dioxygenated by lipoxygenase into hydroperoxy-derivatives. These hydroperoxides serve as substrates for further transformation by different pathways into a series of signaling and defense molecules called phytooxylipins [21,22]. Allene oxide synthase dehydrates the PUFA hydroperoxides to allene oxides, which are further converted to jasmonates [23,24]. Jasmonates are signal molecules leading to the expression of proteinase inhibitors and other foliar compounds with negative effects on herbivore performance [23]. The action of hydroperoxide lyase on PUFA hydroperoxides, on the other hand, leads to the production of a group of specialized metabolites, collectively called “green leaf volatiles” (GLV), which include C_6_ or C_9_ aldehydes, alcohols and their esters [25,26]. An important function of these herbivory-induced GLVs is to prime plant defenses after subsequent herbivory [27]. Young *P. nigra* trees emitted various GLV upon feeding by gypsy moth (*Lymantria discar*). Among these GLVs, (Z)-3-hexenol and (Z)-3-hexenyl acetate were the most abundant [28]. Increased levels of (Z)-3-hexenyl acetate induced the expression of genes involved in the biosynthesis of phytoalexins, a group of anti-microbial compounds, in poplar leaves [29]. Polyketides are widespread among micro-organisms, yet their biosynthesis pathways are less important in the plant specialized metabolism [19]; they, however, do take part in the biosynthesis of some moieties in particular classes of terpenoids, alkaloids and phenolics, e.g., the biosynthesis of stilbenoids and flavonoids (see below).

### 2.2. Terpenoids

**Figure 2:**
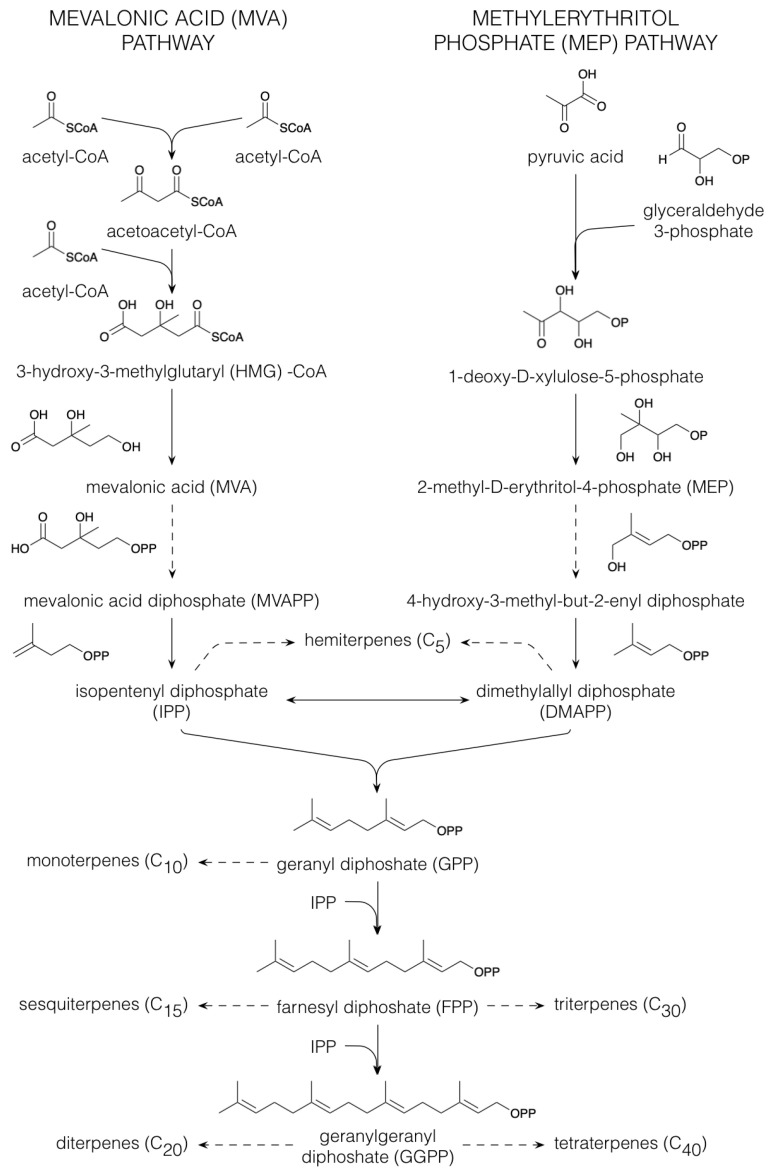
Biosynthesis of terpenoids. Dashed arrows indicate multiple conversions.

Terpenoids comprise the largest group of specialized metabolites in the plant kingdom [30,31,32]. All terpenoids are derived from the five-carbon building blocks, isopentenyl diphosphate (IPP) and dimethylallyl diphosphate (DMAPP), that are derived via either the mevalonic acid (MVA) or the plastidial 2-methyl-D-erythritol 4-phosphate (MEP) pathway [33] (Figure 2). In the MVA pathway, three acetyl-CoA molecules are condensed to form 3-hydroxy-3-methylglutaryl (HMG)-CoA. Following reduction in HMG-CoA to MVA, phosphorylation yields MVA diphosphate (MVAPP), from which a combined decarboxylation and dehydration results in the formation of IPP. Isomerization of IPP provides DMAPP [33]. However, most plant terpenoids are produced via the MEP pathway [34]. Here, decarboxylation of pyruvic acid is followed by a condensation with glyceraldehyde-3-phosphate, yielding 1-deoxy-D-xylulose-5-phosphate. The latter compound is then converted to MEP. Upon activation with cytidine triphosphate, a phosphoanhydride is formed of which the cleavage leads to 4-hydroxy-3-methyl-but-2-enyl diphosphate, which is then the immediate precursor of both IPP and DMAPP [33]. Dephosphorylation of IPP and DMAPP yields the hemiterpenes (C_5_), whereas their concatenation provides geranyl diphosphate (GPP) from which the monoterpenes (C_10_) are derived. The further addition of IPP to GPP produces farnesyl diphosphate (FPP) that is central in sesquiterpene (C_15_) biosynthesis. Linking another FPP to IPP yields then geranylgeranyl diphosphate (GGPP), the precursor of the diterpenes (C_20_). All these coupling reactions occur via a head-to-tail condensation, yet the triterpenes (C_30_) result from joining, tail-to-tail, two FPP molecules. Dependent on the compound class, tetraterpenes (C_40_) are synthesized via tail-to-tail condensation of two GGPP molecules or via head-to-tail condensations [33].

In plants, terpenoids play a role in plant growth and development as photosynthetic pigments (e.g., carotenoids, C_40_), electron-carriers (e.g., ubiquinone, C_40_), regulators of growth and development (e.g., gibberellins, C_20_) or as part of membrane structures (e.g., phytosterols, C_30_) [35,36]. In addition, volatile, low-molecular weight terpenoids (C_5_-C_10_) are involved in the protection of plants against biotic (e.g., microbial pathogens) and abiotic (e.g., thermal stress) stress above- and belowground [37,38]. Furthermore, volatile terpenoids, as constituents of floral scent, play a role in the attraction of plant pollinators [39]. Maize produces a range of non-volatile terpenoids that are elicited in response to biotic attack [40]. For example, phytoalexins, kauralexins and zealexins (C_15_) play a role in maize seedling resistance to *Fusarium verticillioides* [41]. Furthermore, herbivory-damaged maize roots release volatile sesquiterpenes that attract entomopathogenic nematodes which are able to infest the attacking larvae [42]. Herbivore attack of poplar leaves induced the emission of a complex mixture of volatile terpenoids (e.g., (E)-β-ocimene and (E,E)-α-farnescene) and GLVs aboveground [43]. In addition, cockchafer larvae-damaged roots of *Populus trichocarpa* and *Populus nigra* release a mixture of monoterpenes, including β-pinene, camphene and 1,8-cineole [44].

### 2.3. Alkaloids

Alkaloids are characterized by great structural diversity and physiological properties. The sole thing that alkaloids have in common is the presence of a nitrogen atom, mostly present in a heterocyclic ring. This nitrogen atom in alkaloids originates mainly from amino acids, and, in general, the carbon skeleton of the particular amino acid is incorporated in the alkaloid structure, hence, classification is based on their biosynthesis precursor [45]. However, some alkaloid classes derive only their nitrogen atom from an amino acid via a transamination reaction, while the remainder of the structure is derived from, e.g., acetate, shikimate, terpenoids or steroids; these are classified as pseudoalkaloids [45]. Most alkaloids are derived from ornithine (derived from glutamic acid in plants), arginine, lysine, tyrosine and tryptophan. Furthermore, minor alkaloid classes are produced from histidine (imidazole alkaloids), nicotinic acid (pyridine alkaloids), anthranilic acid (quinazoline alkaloids) and xanthosine (purine alkaloids) [45].

**Figure 3:**
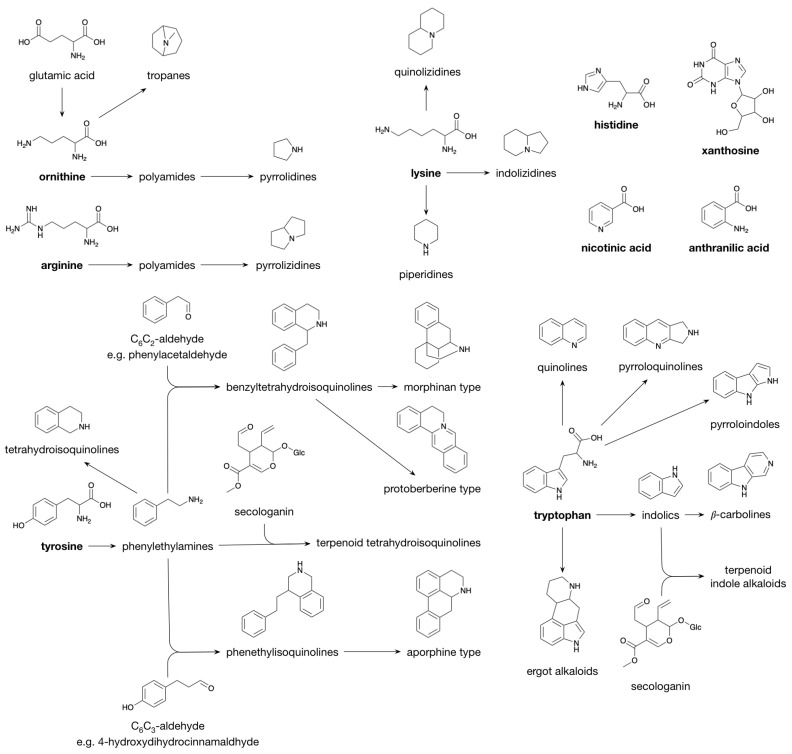
Biosynthesis of Alkaloids.

Polyamines, e.g., agmatine, putrescine and spermidine, are formed from either ornithine or arginine [46]. Polyamines derived from ornithine yield pyrrolidines and tropanes (e.g., cocaine) upon cyclization, whereas cyclization of arginine-derived polyamines yields pyrrolizidines (Figure 3 [45]). Having one additional methylene moiety as compared to ornithine, lysine enters alkaloid biosynthesis pathways that are characterized by similar cyclization mechanisms as those observed for polyamine-derived alkaloids; they lead to the piperidines (six-ring) and quinolizidines (two fused six-rings) rather than the pyrrolidines (five-ring) and pyrrolizidines (two fused five-rings). A third class derived from lysine, the indolizidines, is characterized by a six-ring fused to a five-ring [45]. Similar biosynthetic reaction mechanisms are also observed for the alkaloid classes derived from tyrosine and tryptophan. Decarboxylation of tyrosine and tryptophan leads to the phenylethylamines (e.g., hordenine) and the indolics (e.g., tryptamine). Schiff base formation with a short-chain aliphatic aldehyde/ketone followed by ring closure converts the phenylethylamines and the indolics to the tetrahydroisoquinolines and the β-carbolines, respectively. When Schiff base formation couples a phenylethylamine with a C_6_C_2_ aldehyde, such as phenylacetaldehyde, the benzyltetrahydroisoquinolines (e.g., reticuline) are produced, whereas coupling with a dihydro C_6_C_3_ aldehyde (e.g., 4-hydroxydihydrocinnamaldehyde) or with secologanin produces the phenethylisoquinolines and the terpenoid tetrahydroisoquinolines, respectively. Schiff base formation between indolics and secologanin yields the terpenoid indole alkaloids (e.g., strictosidine). Oxidative radical–radical coupling between the two phenolic moieties present in the structures of benzyltetrahydroisoquinolines and phenethylisoquinolines results in different skeleton types, i.e., the morphinan type (e.g., morphine) and the aporphine type, respectively. Another skeleton type, i.e., the protoberberine type (for example, berberine), is formed from benzyltetrahydroisoquinolines via a second Schiff base formation. Other alkaloid classes that are derived from tryptophan are the quinolines, pyrroloquinolines, pyrroloindoles and the ergot alkaloids [45].

Within plants, alkaloids are believed to serve as a storage form of nitrogen and as protection against herbivory [47]. When a pathogen or predator attacks a plant, the alkaloid can interfere with the predator’s protein synthesis, with the predator’s enzyme activity and/or with the predator’s nervous system [48]. In addition, by inhibiting the growth of competing plants, alkaloids can act as an herbicide [49]. The bioactivity of the alkaloids is due to the presence of the nitrogen atom; the lone pair electrons on the nitrogen can act as proton-acceptors, whereas the hydrogen-atoms in primary and secondary amines can act as a proton-donor. These properties lead to significant improvements in survival rates for plants [48] and, at the same time, provide valuable biological properties for human therapeutics [50]. Plant alkaloids are therefore widely known for their applications as drugs. For example, morphine, an alkaloid originally isolated from *Papaver somniferum* (commonly known as opium poppy), is generally known for the treatment of severe pain. A dominant alkaloid class in monocots, such as maize, are the benzoxazinoids [6,51]. These indole-derived metabolites have been shown to be produced in response to European corn borer (*Ostrinia nubilalis*)-herbivory [52], and inoculation of maize leaves with pathogenic *Curvularia lunata* resulted in the accumulation of HDMBOA-glucoside [53].

### 2.4. Phenolics

**Figure 4:**
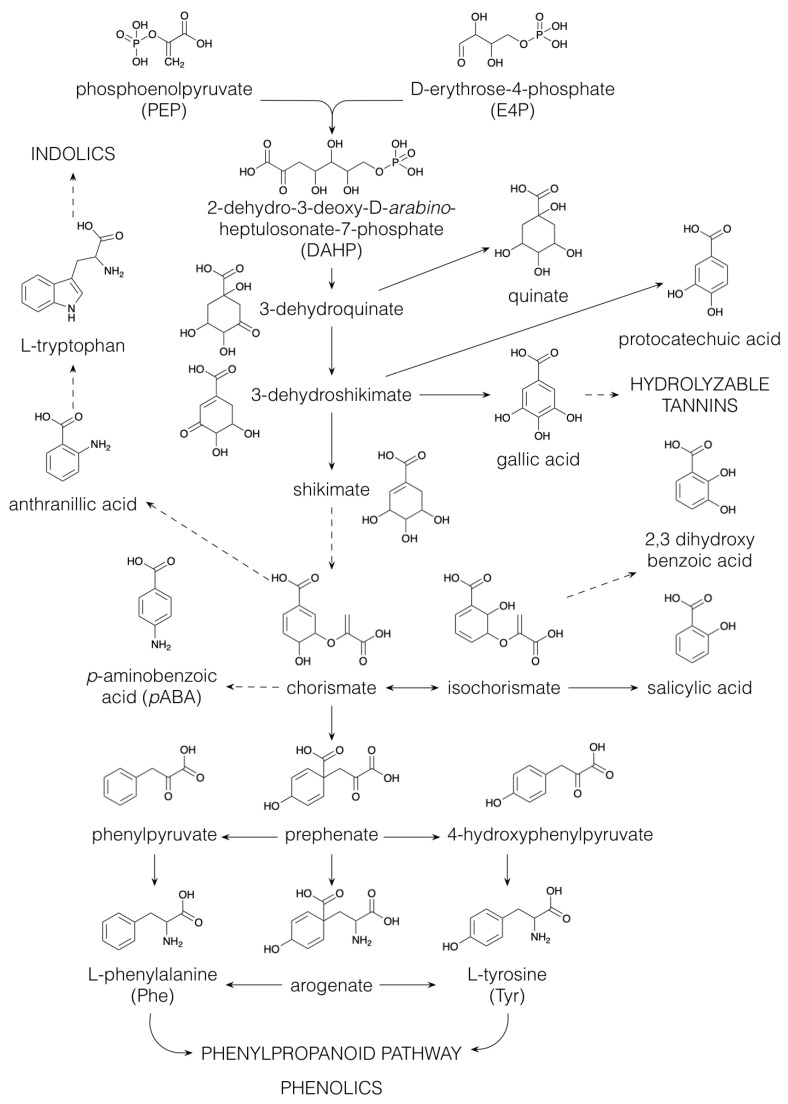
Biosynthesis of the aromatic amino acids via the shikimate pathway. Dashed arrows indicate multiple conversions.

Embryophytes, or land plants, appeared in the mid-Paleozoic era, between 480 and 360 million years ago. The successful adaptation of *Charophyceae* from living underneath the water to living on land was achieved by the production of phenolic compounds, which act as antioxidants in the protection against UV radiation [54]. The classification of phenolics is mainly based on their aromatic skeleton, e.g., C_6_C_3_ (phenylpropanoids), C_6_C_3_C_6_ (flavonoids) or (C_6_C_3_C_6_)_2_ (proanthocyanidins, also called condensed tannins) [54]. There are two biosynthetic pathways leading towards phenolic compounds, namely, via polyketide biosynthesis which mainly occurs in micro-organisms (Figure 1), and via the shikimate pathway (Figure 4), yielding the aromatic amino acids L-phenylalanine (Phe), L-tyrosine (Tyr) and L-tryptophan (Trp) [55]. The shikimate pathway is employed by both micro-organisms and plants but does not exist in humans; therefore, the aromatic amino acids Phe, Tyr and Trp are essential for humans [56]. The shikimate pathway (Figure 4) starts with the aldol condensation of the glycolytic intermediate phosphoenolpyruvate and erythrose-4-phosphate, which is produced via either photosynthesis or the pentose phosphate pathway, to form 2-dehydro-3-deoxy-arabino-heptulosonate-7-phosphate (DAHP). Next, DAHP is converted to 3-dehydroquinate, which can be reduced to quinate or dehydrated to 3-dehydroshikimate, the latter being the precursor of the benzenoids protocatechuate and gallate [55]. Gallate is, in turn, the starter-molecule for the biosynthesis of hydrolyzable tannins [57]. Via shikimate, 3-dehydroshikimate is converted to chorismate, from which some simple aniline derivates are produced using glutamine as an amino-donor, e.g., *p*-aminobenzoic acid (*p*ABA). *p*ABA is part of the folic acid (vitamin B_9_) structure, an essential compound in DNA synthesis, methylation and cellular division [58]. Biofortification of maize by boosting folate biosynthesis has been demonstrated as an attractive strategy to enhance the nutritional value of maize [59,60]. Another aniline derivate, anthranilic acid, is an intermediate in the biosynthetic pathway to the aromatic amino acid Trp, which serves as a precursor for many indolics, for example, indole-3-acetic acid (IAA), a major regulator of plant growth [61]. Isomerization of chorismate to isochorismate is the crucial step in the biosynthesis of the benzenoids, 2,3-dihydroxybenzoate and salicylate (see below). Furthermore, chorismate is an intermediate in the pathway towards the two aromatic amino acids Phe and Tyr. The first step in the biosynthesis of Phe and Tyr involves the conversion of chorismate to prephenate (Figure 4). Then, two alternative pathways are possible. In the first, the arogenate pathway, prephenate, is transaminated to arogenate, followed by a dehydration and decarboxylation to Phe or a dehydration and a decarboxylation to Tyr. In an alternative route, the opposite occurs: prephenate is first dehydrated/dehydrogenated and decarboxylated to phenylpyruvate and *p*-hydroxyphenylpyruvate and then transaminated to produce Phe and Tyr [55]. Most microorganisms utilize the phenylpyruvate/*p*-hydroxyphenylpyruvate pathways, whereas biosynthesis of Phe and Tyr in plants has been predominantly described to occur via the arogenate pathway [62]. However, the alternative biosynthesis of Phe via phenylpyruvate, with Tyr as the amino-donor, has been described in *Petunia hybrida* flowers [63].

**Figure 5:**
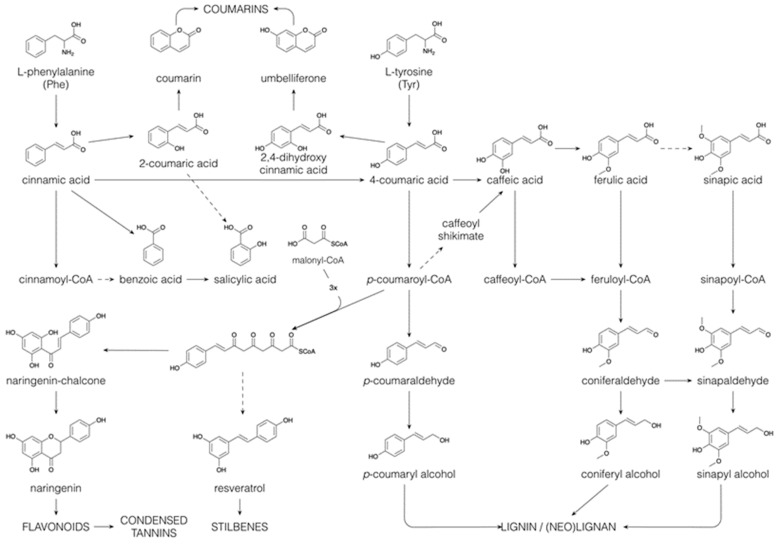
Biosynthesis of coumarins, benzenoids, flavonoids, stilbenoids, lignins and (neo)lignans via the phenylpropanoid pathway. Dashed arrows indicate multiple conversions.

Phe and Tyr form the basis for the C_6_C_3_ phenylpropane unit that is found in a wide range of phenolic compounds, such as phenylpropanoids, flavonoids, coumarins, monolignols, lignins and lignans [64]. The general phenylpropanoid pathway starts with the deamination of Phe leading to *trans*-cinnamate (Figure 5). Successive hydroxylations, by cytochrome P450 monooxygenases, and methylations, by methyltransferases, introduce hydroxyl and methoxyl substituents on the benzene ring, generating *p*-coumaric acid (or 4-coumaric acid), caffeic acid, ferulic acid and sinapic acid [55]. Following CoA esterification, the resulting phenylpropanoyl CoA esters can enter several of the above-mentioned specialized metabolic pathways, such as flavonoid and monolignol biosynthesis (see below). Besides their biosynthesis via the shikimate pathway, benzenoids can be produced from phenylpropanoids by shortening their side chains in a CoA-dependent β-oxidative pathway (analogous to the β-oxidation of fatty acids), or in a CoA-dependent or -independent non-oxidative pathway [65]. The benzenoid salicylic acid can thus be synthesized (i) directly from chorismate, (ii) through isochorismate, (iii) by hydroxylation of benzoic acid (iv) or by side-chain cleavage of *o*-coumaric acid (or 2-coumaric acid), which is formed by the 2-hydroxylation of cinnamic acid (Figure 5, [66]). Salicin, the precursor for the synthesis of acetylsalicylic acid, a widely used pain killer, is produced via reduction in the acidic function of salicylic acid, yielding salicylaldehyde, followed by glycosylation and a subsequent reduction in the aldehyde function to an alcohol [67]. Salicin is the building block of the phenolic glycosides, a major compound class in the Salicaceae [68].

The ortho-hydroxylation of phenylpropanoyl CoA esters is essential in the formation of coumarins. The first step involves a cis–trans isomerization of the side-chain, which is then followed by lactonization [55,69]. As such, cinnamic acid and *p*-coumaric acid give rise to coumarin and umbelliferone, respectively (Figure 5). Further hydroxylation and methylation of the benzene ring yield esculetin and scopoletin, in which the position of the benzene substitutions reflects those found in caffeic acid and ferulic acid.

Reduction of the various phenylpropanoyl CoA esters to their corresponding aldehydes and further to their alcohols constitutes the monolignol biosynthetic pathway. The above-mentioned benzene ring-substitutions may also (partially) occur at the CoA, aldehyde or alcohol level rather than at the acid level [69,70]. Monolignols are exported to the cell wall, where they are oxidized by peroxidases and/or laccases to relatively stable, electron-delocalized radicals, and such monolignol radicals are also thought to be formed by oxidation within the cytoplasm [4,71]. Subsequent radical–radical coupling between monolignols, most often guided in a stereospecific way by a dirigent protein, leads to the synthesis of (neo)lignans. Coupling products derived from two monolignols that are linked via an 8–8 bond are referred to as lignans. If two monolignols are coupled via a different carbon–carbon bond, they are referred to as neolignans, and if they are linked via an ether linkage, they are referred to as oxyneolignans. Sesquineolignans are coupling products between three monolignols, and dineolignans are coupling products involving four monolignols [72]. This structural variety of (neo)lignans is further increased by additional cyclizations, oxydations, hydroxylations and other modifications, resulting in a plethora of structures. Certain lignans, such as podophyllotoxin and its derivatives, have pronounced cytotoxic activity and are used as antitumor compounds [73]. Although the biosynthesis of therapeutic lignans has been studied in depth, the role of these compounds in plants remains obscure today. Some, such as secoisolariciresinol, can serve as antioxidants; however, many others, such as justicidin, are the product of many oxidative reactions and so no antioxidant activity remains [74]. Undirected, non-stereospecific radical–radical coupling between monolignols in the cell wall leads also to the formation of the lignin polymer [75]. Depending on the plant species, the monolignols *p*-coumaryl alcohol (yielding the H-unit in lignin), coniferyl alcohol (G-unit) and sinapyl alcohol (S-unit), and other minor monomers belonging to different phenolic classes (hydroxyarylpropanols, hydroxycinnamyl esters, hydroxycinnamaldehydes, hydroxycinnamic acids, hydroxycinnamate esters, hydroxycinnamides, hydroxybenzaldehydes, hydroxybenzoic acids, hydroxystilbenes and the flavone, tricin), can be incorporated in the lignin polymer, leading to a high, naturally occurring structural variation [69,70]. As lignins from more species and tissues continue to be examined, this structural variability is likely to continue growing. The flexibility of the lignin structure is further shown in mutants and transgenic plants. Upon monolignol biosynthesis pathway perturbation, intermediates accumulate and are converted into a wide range of derivatives, which are partially incorporated into the lignin polymer [7,69,76,77]. Lignin provides the plant with strength to grow upright, and with hydrophobicity for water transport, distributing nutrients from the soil within the plant. Although the incorporated monomers will affect the structure and physico-chemical properties of lignin, a large compositional variation in lignin is allowed without affecting its functionality [69,70,78].

The phenylpropanoyl CoA esters can also serve as starter units for chain extension with malonyl CoA units. Chain extension involving three malonyl CoA molecules occurs most commonly and produces stilbenes (e.g., resveratrol) and chalcones (e.g., naringenin chalcone). Isomerization of the chalcones yields the flavanones (e.g., naringenin). This occurs by a Michael-type nucleophilic attack, yielding a six-membered heterocyclic ring. The flavanones can be converted to a wide range of flavonoids, i.e., flavones, flavonols, flavanols, proanthocyanidins, anthocyanins and catechins, among others, through hydroxylation, reduction and dehydration reactions of the heterocyclic ring. Flavonoids contribute to plant colours necessary for the attraction of pollinators [79]. Chalcones and flavonols account for the yellow colors, whereas anthocyanidins are responsible for red and blue colors and combinations thereof. In addition, flavonoids are involved in plant development regulation, UV protection, plant-microorganism signaling and plant defense [80].

## 3. Structure-Antioxidant Activity Relationships of Flavonoids

Flavonoids, with an estimated 10,000 different members, are ubiquitously present in plants and represent one of the largest groups of specialized metabolites, following the alkaloids (12,000) and terpenoids (30,000) [81]. They possess various biochemical properties, however, the best described property, present in almost all groups of flavonoids, is their antioxidant activity. The antioxidant activity of phenolics is mainly due to their redox properties, which allow them to act as scavengers of reactive oxygen species (ROS), and as singlet oxygen quenchers. In addition, they can chelate metal ions, such as iron or copper, which, otherwise, by the reduction of hydrogen peroxide, could generate the highly reactive hydroxyl radical [13,82,83,84,85]. In a concentration dependent way, flavonoids can also show prooxidant rather than antioxidant activity [86,87]. Because of the distinct antioxidant and prooxidant capacities and structural diversity of flavonoids, this class of polyphenolics has often been the subject of structure–activity relationship studies [86,88,89,90,91].

**Figure 6:**
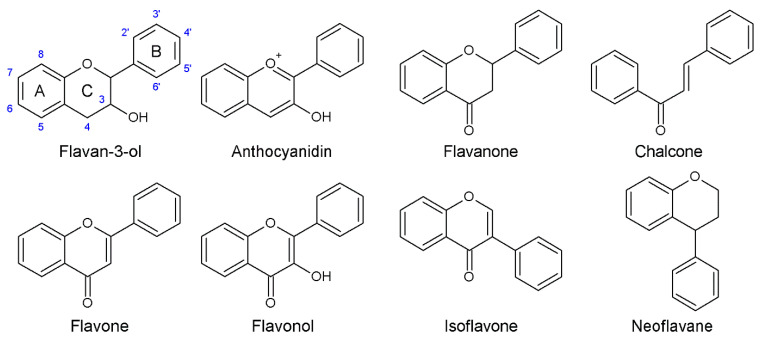
Structures of flavonoids.

The structural variation rises from a few distinct backbone structures (chalcones, flavanols, flavanones, flavones, flavonols, anthocyanidins, isoflavonoids and neoflavonoids) (Figure 6), combined with a wide variety of, predominantly, hydroxylations, methoxylations, prenylation and glycosylations [81]. The backbone does not show any activity [90]. In general, both antioxidant and prooxidant activities increase with the number of hydroxyl substitutions, and ether/acetal bonding (O-methylation or -glycosylation) of the hydroxyl substitution reduces, again, these activities [86,92]. Due to their lower redox potential ($0.2<E_{0}<0.8$), the phenolic hydroxyl groups of flavonoids are able to reduce highly electrophilic free radicals, such as superoxide, peroxyl, alkoxyl and hydroxyl radicals, at diffusion-controlled rates [93]. A strong negative correlation ($R^{2}=0.99$) exists between the redox potential of flavonoids and their antioxidant activity [90]. Phenolic antioxidants can react with free radicals through a hydrogen atom transfer (i), an electron transfer followed by a proton transfer, as has been shown for (+)-catechin and anthocyanidins (ii), or a proton transfer followed by an electron transfer, as has been shown for flavonols (iii) [90,94,95]. In all cases, the scavenging of the radical is thus accompanied by the formation of a flavonoid aroxyl radical [13,96]. Not all –OH substituents play equivalent roles in the redox potential and associated antioxidant activity of flavonoids. In both flavonols and anthocyanidins, the –OH substituent in position 3 of the C-ring has been shown to be a determining factor for the oxidation efficiency [90,91,97]. Delphidin, which carries an –OH substituent at position 3 of the C-ring, on top of three –OH substituents on the B-ring and two –OH substituents on the A-ring, has a very low redox potential ($E_{0}^{\prime }=0.29\;V$) and is therefore easily oxidized. Delphinidin-3-O-glucoside, which still carries a total of five –OH substituents but, where the 3–OH substituent is blocked by an ether bond to the carbohydrate, has a redox potential of 1.25V, similar to a flavone devoid of any –OH substituents [90]. Nevertheless, the oxidation of flavonols and anthocyanidins is thought to start by losing a proton and an electron (or an electron and a proton) in an –OH substituent of the B-ring, and not at the 3–OH location of the C-ring. It was proposed that the key role of the 3–OH substituent in the oxidation efficiency of flavonols and anthocyanidins is related to its effects on the distribution of the electron density of the neutral molecule [90]. If the 3–OH substituent is free, the redox potential of flavonols and anthocyanidins further decreases, and radical scavenging capacity concomitantly increases, with the number of hydroxyl substituents on the B-ring. Even the effect of the oxonium ion in the core structure of anthocyanidins, leading to a higher redox potential than the more electron-rich oxygen-ether in flavonols, is annihilated by increasing numbers of –OH substituents on the B-ring. For example, the redox potential of kaempferol is much lower than the redox potential of pelargonidin (both carrying a single –OH group on the B-ring); however, myricetin and delphinidin (both carrying three –OH groups on the B-ring) have similar redox potentials [90]. In flavones, which lack a 3–OH group, the most reactive hydroxyl group is the 7–OH group, situated on the A-ring [13].

The efficiency of flavonoids as antioxidants depends not only on the radical scavenging capacity, but also on the stability of the radical formed after scavenging. Highly reactive secondary radicals would propagate a chain reaction rather than interrupting it [84]. A measure of the reactivity of radicals is their decay rate in a second-order dismutation (also called disproportionation) process, in which two radicals convert to one compound of higher and one of lower oxidation state. The stability of flavonoid radicals is expected to increase with the extension of the conjugated system. Three structural groups are determining for the radical scavenging and/or antioxidative potential of flavonoids [84]. First, a 3′,4′-dihydroxy (catechol) structure of the B-ring, as in epicatechin, rutin or quercetin, is a major radical target site, and participates in electron delocalization, conferring a higher stability of the aroxyl radical. The aroxyl radical formed is, in that case, an *o*-semiquinone ($Q^{•-}$). Flavonoids that lack the catechol structure, even if they can be highly efficient radical scavengers such as kaempferol, decay too fast to be potent antioxidants [84]. The determining role of the catechol structure for antioxidant activity has similarly been observed in lignans. Lignans with catechol moieties exhibit the highest radical scavenging capacity, whereas the corresponding guaiacyl (i.e., 3-methoxy-4-hydroxyphenyl) derivatives show a slightly weaker scavenging capacity [98]. A second determining structural group is the 2,3-double bond in conjugation with a 4-oxo function. This combination allows tautomerization of the aroxyl (*o*-semiquinone) radical into a semiquinone methide, which results in a conjugated system involving all three ring systems (A, B and C). Such extensive electron delocalization provides extra stability to the aroxyl radical [84]. For example, especially in monocots, the flavonol tricin, which does not contain a catechol B-ring but does contain the 2,3-double bond in conjugation with a 4-oxo function, is oxidized in the cell wall and used as a monomer during lignin polymerization [99]. Finally, the third important functional group is the additional presence of 3– and 5–OH groups in the A-ring [84]. All polyphenol *o*-semiquinone radicals can decay by bi-molecular disproportionation to form the more stable *o*-quinone (Q) and the parent hydroxy compound, as follows in the case of quercetin [87]:

(1) $$2Q^{•-}\to Q+quercetin$$

The 3– and 5–OH groups in the A-ring allow tautomerization of o-quinone into three quinone methides and contribute to an increased radical-scavenging potential [84,88]. The tautomerization depends on the rearrangement of the 3–OH group on the heterocycle (the C-ring) to form a 3-keto group. Lack of the 3–OH thus prevents quinone methide isomerization. The availability of the 3–OH group, however, also depends on the presence of a 5–OH group on the A-ring. In flavonols lacking a 5–OH, the 4-keto group forms a hydrogen bond with the 3–OH group, making the latter unavailable for tautomerization to a 3-keto group. A 5–OH group, however, forms an alternative hydrogen bond with the 4-keto group, leaving the 3–OH group more available for tautomerization [88]. The presence of both 3- and 5–OH groups enables, thus, the formation of stable quinonic structures upon flavonoid oxidation. Interestingly, the catechol structure on the B-ring and the 4-oxo function, in combination with the 3– and 5–OH groups, not only improve the ROS scavenging capacities of flavonoids, but confer also their metal chelation capacities, as they form binding sites for metal cations [13]. In summary, flavonoids carrying at least two *o*-hydroxyl groups on their B-ring are easily oxidized to quinones by ROS, and their antioxidant capacity is further improved by the presence of a 2,3-double bond, a 4-carbonyl function and 3– and 5–OH groups. Flavones lacking the catechol system exhibit generally weak antioxidant potential because oxidation leads to the formation of unstable radicals that would rather propagate a chain reaction instead of interrupting it [84,89,100]. Flavonoids with a phenol-type B-ring, however, have a greater ability to absorb UV-wavelengths than flavonoids with a catechol-type B-ring, indicating that, at least to some extent, structural diversity correlates with functional diversity [101].

Under certain conditions, flavonoids also act as prooxidants, and many different mechanisms have been proposed to explain this phenomenon [102]. Several studies have shown that the prooxidant activity is related to the same functional groups as the antioxidant activity, with quercetin being a strong prooxidant. For example, in human lymphocytes, the generation of superoxide anion radical and products of lipid peroxidation increased with increasing concentration of quercetin, hesperetin or naringenin, and this effect was associated with increasing aroxyl radical formation [103]. The effect of quercetin was much stronger than that of the flavanones hesperetin and naringenin, and this was attributed to the *o*-semiquinone and *o*-quinone oxidation products, typical for catechol carrying compounds. Quercetin-derived *o*-semiquinones and quinones can undergo (futile) redox cycling, with superoxide formed in a pro-oxidant effect [87]. For example, $Q^{•-}$ can initiate redox cycling by reacting with O_2_ to form superoxide and Q [87]:

(2) $$2Q^{•-}+O_{2}\to Q+O_{2}^{•-}$$

Moreover, *o*-semiquinone and quinone type metabolites may act as electrophiles, forming covalent adducts with DNA, and are therefore mutagenic [88,104].

Some of the strongest antioxidants in plants are catechins and proanthocyanidins, although they do not fulfill the above criteria [105]. Catechins are flavan-3-ol derivatives and lack the 2,3-double bond, allowing four possible stereoisomers, of which (+)-catechin and (-)-epicatechin are the most common [105]. Catechins carry a catechol B-ring, whereas gallocatechins carry a pyrogallol B-ring (3′– 4′–, and 5′–OH groups). Moreover, a gallate (G)-ring is often added to catechins or gallocatechins by the esterification of gallic acid with the 3–OH group of the C-ring [85]. Since they lack a 2,3-double bond and often have an esterified 3–OH substituent, the strong antioxidant activity of catechins cannot be attributed to the structural groups mentioned above. Instead, the antioxidant capacity of catechins and their derivatives is directly related to their capacity to multiply the number of neighboring hydroxyl groups within the same molecule [105]. Both the catechol and pyrogallol groups (B- and G-rings) are potential sites for radical attack. The scavenging rates for gallate esters exceed those of diffusion-controlled reactions, because of the presence of several radical target sites within the same molecule (B- and G-rings) [105]. Similarly to flavonols, disproportionation of the aroxyl (semiquinone) radicals results in the formation of a quinone structure [105]. The semiquinones and quinones formed from proper flavonols behave, however, distinctly from those derived from flavan-3-ols. Those of catechins (flavan-3 -ols) preferentially oligomerize via phenolic coupling reactions. Because each monomer retains its original number of hydroxyl groups, the antioxidant capacity increases with the degree of polymerization until the oligomers become insoluble and precipitate [106,107,108]. The superior antioxidant potential of proanthocyanidins, as compared to flavonols, resides thus in their polymerization and concomitant increase in radical target sites.

The antioxidant activity of flavonoids comprises the scavenging of different reactive oxygen species (ROS), including singlet oxygen. Singlet oxygen, abbreviated as ^1^O_2_, is not a radical but an excited state of oxygen, and represents the major ROS involved in photooxidative damage to plants [109]. Specific comparative activity studies have focused on the structure–function relationship between flavonoids and ^1^O_2_ quenching. The quenching can be a chemical process, in which the quencher is combined with oxygen or is oxidized, or a physical process, in which the quencher enters an excited vibrational or electronic state. Chemical quenching of ^1^O_2_ by flavonoids depends largely on the structure of ring C. The presence of a C2-C3 double bond in ring C in flavonols and flavones allows cycloaddition of singlet oxygen to this double bond, finally leading to the opening of ring C. In flavonols, this chemical reaction with singlet oxygen is further accelerated by the presence of 3–OH substitution on ring C [110]. Hexosylation of this 3–OH decreases the reactivity [110]. On rings A and B, OH substituents do not affect the chemical reactivity of flavonols and flavones toward singlet oxygen, although O-alkyl sutbstituents on these rings reduce the activity, probably due to steric hindrance. Flavanones and flavan-3-ols (catechins), in contrast to flavonols and flavones, miss the C2-C3 double bond in ring C and are therefore chemically inert towards ^1^O_2_ [110]. The quenching activity of most flavonoids is, however, dominated by the physical process, and the efficiency of this physical process mainly depends on the presence of a catechol (B)-ring (3′– and 4′–OH groups on ring B) or a pyrogallol (B)-ring (3′–, 4′–, and 5′–OH groups on ring B) [85,110]. The pyrogallol (B)-ring, present in gallocatechin, confers even stronger ^1^O_2_ quenching activity than a catechol (B)-ring [85]. Catechin and epicatchin, which contain a catechol (B)-ring, are much stronger physical quenchers of singlet oxygen than flavonols, flavones or flavanones containing the same catechol (B)-ring, and this higher efficiency has been attributed to the absence of a carbonyl group on ring C, leading to a less planar structure of catechins [110]. Furthermore, addition of a gallate (G)-ring in catechins or gallocatechins by esterification of gallic acid with the 3–OH group of the C-ring contributes remarkably to the quenching of ^1^O_2_. This increase in quenching activity surpasses, by far, the quenching activity of the G-ring itself. It has therefore been proposed that the conformation of the G-ring containing catechins leads to electronic π−π interaction between B- and G- rings, lowering as such the redox potential of the galloylated molecules and increasing the total electron-donating capacity [85]. It should be noted that this conformation and electronic π−π interaction have not been confirmed in a crystal structure to date. Overall, it can be concluded that, even if essentially antioxidant capacity of flavonoids resides in their phenolic hydroxyl groups, different antioxidative mechanisms can be involved for a single flavonoid structure, and distinct flavonoid structures show distinct antioxidant mechanisms.

A large portion of the structural diversity among polyphenolics results from oxidative coupling reactions, triggered by initial radical scavenging reactions. Combinatorial coupling between a flavonoid semiquinone radical and a phenylpropanoid radical, such as coniferyl alcohol, forms a flavonolignan [111,112]. Combinatorial radical–radical coupling among flavones, flavonols, dihydroflavonols, flavanones, isoflavones, aurones and chalcones forms bi-, tri-, and tetraflavonoids [113,114,115,116,117,118,119,120,121]. Because the radical can be delocalized along the flavonoid conjugated system, various C–C or C–O–C linkage types are produced. These flavonoid oligomers can be distinguished from proanthocyanidin oligomers by the coupling mechanisms (proanthocyanidins are coupled through nucleophilic attacks) and by their monomers (proanthocyanidins are coupling products of flavan-3-ol monomers); however, the distinction is somehow arbitrary because “mixed” dimers, for example, flavan-3-ol → dihydroflavonol, and “nonproanthocyanidins” comprising oxidatively coupled flavan-3-ols have also been identified [113]. Similarly to proanthocyanidins, dimer or oligomer molecules may exhibit enhanced activity relative to the simple monomer units. For example, bikaempferol showed stronger ROS scavenging activity than kaempferol [121]. This amplifying effect can be induced by the increased number of phenolic hydroxyl groups and by the extension of the conjugated system over the two molecules. Not all plant extracted biflavonoids, however, exhibit strong antioxidant activity [120]. In certain cases, dimerization may have led to new functionalities. Biflavonoids have been mainly studied for their strong cytotoxic activity, suggesting a defensive role against herbivores in plants [119,122,123]. It is not known if the coupling occurs in a directed way, guided by a dirigent protein, in analogy with lignan biosynthesis, and also demonstrated for the synthesis of the phenolic terpenoid (+)-gossypol in cotton (*Gossypium* sp.) [124,125]. In certain cases, however, racemic mixtures of biflavonoids have been reported, indicating an uncontrolled, non-stereospecific radical–radical coupling [120]. Undirected radical–radical coupling in an oxidative cell environment could terminate radical chain reactions. The coupling products would then be, in some way, waste products with highly variable structures but, a priori, no proper function. In poplar, under oxidative conditions, monolignol radical coupling occurs within the cytoplasm [4,71]. Phenylcoumaran benzylic ether reductase (PCBER), an extremely abundant enzyme in poplar wood, reduces various products that arise from the oxidative coupling of monolignols, regenerating, as such, radical scavengers in an oxidative intracellular environment [71]. Similar recycling loops of other types of polyphenolic radical coupling products could be highly efficient contributors of radical scavengers in plant cells, although confirmation of this functionality awaits, today, the functional characterization of more PCBER-like reductases in the plant specialized metabolism.

## 4. Decoration and Conjugation in Specialized Metabolism

### 4.1. Diversity of Decorations

The majority of the structural diversity in the plant specialized metabolome arises from “decorative” biosynthetic steps, such as hydroxylations, methylations, glycosylations, acylations and prenylations [126]. The number of different modifications is rather limited and the nature of the decorations for different classes of specialized metabolites is often the same. For example, classes as far apart as flavonoids, indole alkaloids and apocarotenoids are all decorated with glucose and with malonylglucose [1,3]. In the specialized metabolite profile of a plant extract, the common decorations are reflected in an overrepresentation of certain mass differences corresponding to the difference between two core structures. For example, in the maize specialized metabolome, the mass difference of 108.021 Da, corresponding to the difference between a phenylpropanoid and a flavonoid core structure, is observed between *p*-coumaric acid and naringenin, but also between caffeoyl hexose and eriodictyol 7-O-hexoside (hydroxylation and addition of a hexose to both) or between sinapoyl hexose and methoxy-homoeriodictyol (two methoxylations of both), as well as between dihydroxyindole-3-acetic acid (caffeoyl) hexoside and dihydroxyindole-3-acetic acid (eriodictyol-O-) hexoside (hydroxylation and addition of dihydroxyindole-3-acetic acid and hexose to both) [6]. The majority of plant specialized metabolites are glycosylated and/or acylated, with acylations being aromatic (*p*-coumaric, caffeic, ferulic, sinapic, gallic or *p*-hydroxybenzoic acids) or aliphatic (malonic, acetic, malic, succinic or oxalic acids). Because of the multitude of different possible decoration sites (hydroxyl groups), and the multitude of possible combinations of decorations, the number of possible structures grows exponentially with each added decoration [127]. Moreover, N-acylation of alkaloids, such as anthranilic acid or polyamines, leads to the formation of a high variety of hydroxycinnamic acid amides [128]. Intramolecular cyclization through phenol-oxidative coupling between the phenolic rings of two *p*-coumaroyl substitutes in bis-(*p*-coumaroyl) polyamines is the biosynthetic mechanism leading to cyclic alkaloids such as lunarine (in *Lunaria annua* seeds) and aphelandrine (in *Aphelandra* sp. roots) [129,130]. The introduction of phenolic rings through aromatic acylation allows, thus, further structural diversification through oxidative coupling.

### 4.2. High Molecular Weight Conjugates

Glucosylation and acylation allow for the concatenation of several specialized metabolites, sometimes of different metabolic classes. In the maize specialized metabolome, phenylpropanoid glycosides are coupled to benzoic acids, flavonoids, phenylethanoids and indolics [6]. Hexosylated flavonoids, but also free hexoses or dihexoses, often carry multiple aromatic acylations, often of different natures [3,6,131]. Less frequently, the hexose moiety in poly-acylated aromatic conjugates is replaced by an alternative polyol, such as glucaric acid [2]. The feruloyl moieties, both in glucaric acid and hexose conjugates, are sometimes coupled by radical–radical coupling to mono- or oligolignols, allowing these coupling products to be stored as such in the leaf vacuole [2,4]. In contrast to glucaric acid conjugates, where the two carboxylic acid groups of glucaric acid remain unesterified, other aromatic conjugates involve esterification to the carboxyl groups of dicarboxylic acids such as succinic acid or 3-hydroxy-3-methylglutaric acid. For example, in blue Agapanthus flowers, a *p*-coumaroylated delphinidin diglycoside is attached to a favonol triglycoside via a succinic acid diester link [132]. In this conjugate, the aromatic acyl group and the colourless flavonoid both stabilize the blue color of the anthocyanin delphidin diglycoside. Esterification involving the dicarboxylic acids 3-hydroxy-3-methylglutaric acid (HMGA) and succinic acid allowed the formation of a macromolecular polyester structure carrying glucosylated lignans, glucosylated flavonoids, phenylpropanoic acids and ferulic acid coupled oligolignols in the outer integument of flax seeds [127]. More common polyesters in higher plants are cutin and suberin, of which the latter contains aromatic domains derived from cinnamic acids [133]. Cutin and suberin function as protecting barriers against desiccation or biotic and abiotic stresses.

### 4.3. Function of Decorations

An excellent review recently discussed the current knowledge of the biological function of flavonoid hydroxylation, methylation, glycosylation and acylation [134]. Glycosylation and aliphatic acylation are generally seen as strategies to detoxify or increase the solubility of specialized metabolites or to change their biological activity. Regulatory functions of acylations have also been demonstrated. For example, malonylation of flavonoid glucosides increases the transport efficiency of flavonoid glucosides into the vacuole, indicating that acylation participates in the regulation of metabolic homeostasis [135,136]. In phenylpropanoid biosynthesis, *p*-coumaroyl shikimate is preferred over *p*-coumaric acid as a substrate for 3-hydroxylation by *p*-COUMARATE 3-HYDROXYLASE (C3H), and the product, caffeoyl shikimate, is subsequently hydrolyzed back to the carboxylic acid level (caffeic acid) (Figure 5) [137,138,139]. The occurrence of shikimate ester intermediates represents an energy consuming biosynthetic detour, and has been proposed to play a regulating role in the phenylpropanoid biosynthesis pathway [69].

Regarding biological activity, aromatic acylation of glycosylated flavonoids enhances UV absorption, and it has been proposed that this results from an intramolecular co-pigmentation-like effect, due to the interaction between the aromatic A-ring of the flavonoid and the phenolic ring of the acyl [134]. Depending on the glycosylation site and the anthocyanidin type, glycosylation can decrease, leave unchanged or increase the antioxidant activity of anthocyanidins [140]. The above-described structure–function relationships of undecorated flavonols and anthocyanidins predict a similar antioxidant activity of these two categories, or a slightly stronger effect of flavonols, in plants. However, it has been repeatedly shown that, in plant extracts, anthocyanins contribute more to antioxidant activity than flavonols [141,142]. In berries and small fruits (raspberry, blackberry, sour cherry, strawberry, chokeberry, elderberry and blueberry), antioxidant activity was more strongly correlated with total anthocyanin content (r = 0.99) than with total flavonols, or total quercetin derivatives (r = 0.93) [141]. In the fern *Pteris vittata*, subterranean parts have stronger antioxidant activity than aerial parts and the main difference in the metabolomes of these two parts resides in the presence of anthocyanins in the subterranean parts that were undetected in aerial parts [142]. These observations emphasize that functional studies of aglycones need to be relativized and that the final activity of the specialized metabolome is determined by the decorations. It should also be noted that studies relating antioxidant activity of plant extracts to their metabolome, published today, are almost always biased by the targeted metabolite profiling methods on which they are based. A plethora of structures is present in the specialized plant metabolome, of which the majority has never been described, is not included in databases and is hard to detect by targeted approaches. Detection and characterization of biochemically related compounds can be aided by untargeted approaches via the construction of molecular networks. In this approach, pairs of features (ionized metabolites detected by LC-MS) are connected based on mass spectral similarity or based on mass differences that potentially represent well-known decorations or biotransformations [3,143]. Only a wider coverage of the specialized metabolome, including the modifications and decorations that were not recorded in databases, will allow more reliable studies of its functioning.

The decorations are subject to dynamic change during growth and development of the plants or in response to environmental stimuli [73]. In *Linum album* hairy root culture, during the first days of the exponential phase, 6-methoxypodophyllotoxin glucoside is deglucosylated, whereas, after the exponential phase, when the total tissue weight remains stable, 6-methoxypodophyllotoxin is glucosylated again [73]. In Arabidopsis, kaempferol and quercetin 3-O-β-glucoside-7-O-α-rhamnoside accumulate in response to abiotic stress, whereas the disappearance of the stress induces enzyme-mediated cleavage of the 3-O-β linked glucose moiety of these flavonoid biglucosides, releasing 7-O-α-rhamnosides [144]. Exudation of fragments of the flax seed macromolecular polyester early during germination suggested the activity of hydrolytic enzymes, liberating large amounts of phenolics in the spermosphere [145]. Such observations indicate that metabolite modification, allowing rapid sequestration or release of active compounds, is a key element in the adaptive capacity of plants to changes in their environment [126]. Strong correlations between individual metabolites and antioxidant activity of plant extracts are rarely observed, even if multivariate models can show the predictive power of the metabolome for antioxidant activity and can indicate which groups of metabolites contribute most to this activity [142]. It would be interesting to investigate further if decorations by common glycosylations and/or acylations, conjugation and polyester formation, in combination with enzymatic hydrolysis, form a tool of the plant specialized metabolism for reversible storage and mass release of high concentrations of synergistically functioning metabolites.

### 4.4. Decoration Pathways

In order to better understand the role of specialized metabolite modifications and conjugation, and the control mechanisms governing those, it can be of interest to know the order of the different modifications leading to the complex compounds. Large scale untargeted metabolite profiling by LC-MS or GC-MS, whether or not in combination with the construction of a mass difference network, a spectral similarity network, a structural similarity network or an abundance-based correlation network, may reveal potential pathway intermediates and be a first step in the reconstruction of specialized metabolite modification pathways [143]. For example, in the tropane alkaloid spectrum in *Datura innoxia* (*Solanaceae*), among the high diversity of simple and multiple esters of tropine or pseudotropine, no correlation clustering of tropine or pseudotropine esters with common acyl moieties (for example: all tigloyl esters) was observed, suggesting that the availability of acyl donors is not the main or only factor determining the accumulation of the esters. Instead, robust correlations suggested that 3α-tigloyloxy-6-propionyloxy-7-hydroxytropane is formed by 3α-hydroxylation of tropine, followed by 3α-O-tigloylation, 6-hydroxylation, 6-O-propionylation and, finally, 7-hydroxylation. The fact that alternative precursors, such as 3α -hydroxy-6-propionyloxytropane, were not detected corroborated this order of modifications [146]. In general, knowledge about the presence or absence of specific metabolites in a particular plant organ is a crucial first step in the construction of putative pathways, whose relevance can then be further tested by gene function analysis [6].

## 5. Plasticity and Promiscuity of the Plant Specialized Metabolism

Compared to enzymes of the primary metabolism, enzymes of the specialized metabolism are, paradoxically, less specialized. They have lower *k*_cat_ values (the turnover number) and a broader substrate specificity, meaning that they catalyze the same reaction at similar, relatively low, rates on multiple substrates [15,147]. If the different substrates for low substrate specific enzymes are available, this may result in pathways with a grid-like structure, as is commonly seen in the biosynthesis pathways of the core skeletons of specialized metabolites, including monolignols, flavonoids, oxylipins and brassinosteroids [148,149]. Poor substrate specificity of modifying, rather than the core-pathway enzymes, may contribute extensively to structural diversity, depending on the availability and compartmentation of the substrates. The broad substrate specificity of BAHD acyl transferases is well documented [150,151]. Across the Solanaceae family, considering 12 aliphatic CoA donors with only a few BAHD enzymes as the catalysts, more than 6000 theoretical structures for tetra-acylsugars are possible [152]. Broad specificity also leaves the door open for substrate promiscuity of the enzymes, referring to their ability to catalyze slow, secondary, physiologically irrelevant reactions on alternative substrates that become available under particular conditions [15,153]. Promiscuous activity becomes apparent in mutants and transgenic plants with perturbed biosynthesis pathways. For example, *Arabidopsis coumarin synthase* (*cosy*) mutants accumulate a large array of *o*-hydroxyphenylpropanoids that are not detected in wild type plants [5]. The combination of a mutation in the gene encoding caffeic acid *O*-methyltransferase (comt) with over-expression of ferulate 5-hydroxylase in Arabidopsis resulted in shifts in phenolic metabolism toward the formation of 5-hydroxy-substituted phenylpropanoids. In the latter example, it was proposed that these compounds originated from existing biochemical routes that are normally used for 5-methoxylated and 5-unsubstituted phenylpropanoids [76]. In general, perturbation of an enzymatic step in the phenylproponoid metabolism leads to diversion of the metabolic flux into upstream and branch pathways, and sometimes the newly accumulating phenolic molecules oxidatively couple into small oligomers, further extending the structural diversity [69]. In poplar, down-regulation of the gene encoding CINNAMYL ALCOHOL DEHYDROGENASE1 (CAD1), an enzyme that catalyzes the last step of monolignol biosynthesis, led to, besides the accumulation of a range of hydroxycinnamate derivates that were not observed in wild type plants, the apparition of a newly identified coupling product of two sinapaldehyde radicals [7]. In Arabidopsis *atr2* mutants (*arabidopsis thaliana cytochrome p450 reductase2*), radical coupling lead to the formation of a series of new, H-unit enriched oligolignols [77]. Also in the primary metabolism, substrate promiscuity and catalytic promiscuity, i.e., an aberrant reaction catalyzed on the enzyme’s normal substrate, are a common phenomenon [126,154]. Recent studies show that products formed by mis-catalysis in the plant primary metabolism are detoxified by repair enzymes that reconvert the abnormal metabolites to normal ones [154,155,156,157]. In plants with perturbed phenylpropanoid biosynthesis, modifying enzymes transfer hexose, acetylhexose, sulfate, hexuronate, malate, glutamate, aspartate, shikimate or quinate to abnormally accumulating phenolics, altering their solubility, toxic potential and compartmentalization [69]. The combination of oxidative radical coupling and several enzymatic decorations can result in highly conjugated molecules, such as, for example, a radical coupling product of coniferyl alcohol and ferulic acid, conjugated with a glucose and a malate moiety, identified in *ccr1* (*cinnamoyl-coa reductase1*) Arabidopsis mutants [158]. The physiological consequences of accumulating products of enzyme promiscuity in the specialized metabolism remain largely unknown. They have been proposed to contribute to the dwarfed phenotypes observed in many lignin mutants [69]. Nevertheless, there is a growing belief that the plant specialized metabolism tolerates and even promotes enzyme promiscuity [15,153]. Promiscuity as an inherent element of the plant specialized metabolism implies the tolerance of apparently wasted flux into a variety of metabolites that can reach relatively high complexity, but of which the formation has not been positively selected for by evolution. However, promiscuous activities increase the chances of producing new chemicals, potentially contributing to the fitness of the plant under new environmental challenges and can provide the starting point for evolution toward new enzyme functions and metabolic pathways [15,149,153,159].

## 6. Conclusions

At its basis, the specialized plant metabolism is not extremely diverse. Four major pathways, each starting from a few distinct primary metabolism precursors, lead to distinct basic carbon skeleton core structures: polyketides and fatty acid derivatives, terpenoids, alkaloids and phenolics. Structural diversity in the specialized metabolism, however, expands exponentially with each subsequent modification of the core structure, decoration and conjugation. Series of subsequent conjugations among decorated specialized metabolites lead to the formation of polyglycosides and polyesters in rare cases of macromolecular weight. Many specialized metabolites are involved in redox reactions, and part of the structural diversity can also be attributed to follow-up reactions among oxidized structures, leading to the extremely diverse array of biflavonoids, (neo)lignans, oligolignols, proanthocyanidines and phlobaphenes, and derivatives of aromatically decorated compounds. A detailed analysis of the relation between structure and antioxidant activity of flavonoids shows that core structure, functional groups and degree of polymerization all affect the activity of a compound, and these activities are further modified by the subsequent decorations and conjugations. Nevertheless, the hypothesis that a distinct biological function can be attributed to each distinct structure is difficult to keep up. This impression reaches its paroxysm if one thinks about the specialized metabolomes of mutants or transgenic plants in which a biosynthetic step has been boosted or suppressed artificially. The plasticity of specialized metabolism makes it so that simple modifications often lead to the formation of a whole series of new metabolites, to which a function, optimized by evolutionary constraints, cannot be attributed. Lignin polymers derived from alternative monomers, which are formed in transgenic plants or mutants with perturbed monolignol biosynthesis, often remain perfectly functional. This suggests that functional constraints are limited to certain essential parts of the core structures and/or functional group (s), leaving the doors open for evolution toward wide structural diversity. Instead of focusing on the function of individual structures, one can also question what the roles of more general aspects of the full specialized metabolome are, such as the role of the structural diversity itself, or of conserved functional groups, the plasticity and adaptability of the biosynthesis pathways, the total quantity of specialized metabolites, the compartmentalization or conjugation and hydrolysis, and the control mechanisms of all these aspects. A prerequisite for a real study of how the plant specialized metabolism functions as a whole, is that we can relatively easily identify, and to some extent also quantify, a large percentage of the metabolome, present under different environmental or developmental conditions, in different tissues of different species. A high-throughput technique similar to what RNAseq or microarrays represent for transcriptomics, is far from the reality today in the field of metabolomics. However, with the recent concerted efforts of analytical chemists (GC-MS, LC-MS, NMR), computational biologists, computer programmers and database developers, automated annotation and structural characterization of unknown metabolites is steadily making progress. As the Buddhist poet Issa (1772–1858) probably would say: “Also the snail climbs mount Fuji, but slowly, slowly”.
